# Protective effect of astaxanthin and metformin in the liver of rats in which the polycystic ovary syndrome model was formed by giving letrozole

**DOI:** 10.22038/IJBMS.2023.68032.14872

**Published:** 2023

**Authors:** Tuğba Taştan Bal, Nurhan Akaras, Özlem Demir, Rüstem Anıl Ugan

**Affiliations:** 1 Department of Histology and Embryology, Faculty of Medicine, Erzincan Binali Yıldırım University, Erzincan, Turkey; 2 Department of Histology and Embryology, Faculty of Medicine, Aksaray University, Aksaray, Turkey; 3 Department of Pharmacology, Faculty of Pharmacy, Ataturk University, Erzurum, Turkey

## Abstract

**Objective(s):**

In this study, the effects of astaxanthin on liver tissue in rats with polycystic ovary syndrome (PCOS) were evaluated.

**Materials and Methods:**

Fifty-four Spraque-Dawley rats were divided into 9 groups: Groups: Control, PCOS, PCOS+Metformin (Met), PCOS+ Astaxanthin (ASX)10, PCOS+ASX20, PCOS+ASX40, PCOS+Met+ASX10, PCOS+Met+ASX20, and PCOS+Met+ASX40. PCOS was induced in female rats by oral administration of letrozole (1 mg/kg) for 21 days. Rats were treated with ASX (10 mg/kg), ASX (20 mg/kg), ASX (40 mg/kg), and metformin (20 mg/kg) for 7 days after PCOS induction. At the end of the experiment, malondialdehyde (MDA) and superoxide dismutase (SOD) levels were measured in the liver tissue. The liver was stained with hematoxylin/eosin for histological examination. Additionally, NF-kB and caspase 3 were analyzed immunohistochemically.

**Results:**

A remarkable abnormality was observed in the biochemical and histological parameters in the liver tissue of the PCOS model rats. Astaxanthin dose-dependently normalized the MDA level. Additionally, astaxanthin showed a protective effect by increasing the SOD level and increasing its antioxidant activities. We observed that administration of astaxanthin in addition to metformin applied in the standard was more effective. Caspase 3 and NF-kB immune positivity was lower in the groups given astaxanthin compared with PCOS. Histologically, it was observed that astaxanthin improved the deteriorated liver morphology in the letrozole-induced PCOS group.

**Conclusion:**

According to our results, it was observed that astaxanthin had antioxidant, anti-inflammatory and anti-apoptotic effects on PCOS in the treatment groups. Therefore, it was concluded that astaxanthin may have a protective effect against PCOS side effects.

## Introduction

Polycystic ovary syndrome (PCOS) is very common among women and is characterized by hypothalamic-pituitary-ovarian axis dysfunction and anovulation (1). PCOS has significant effects on obesity, insulin resistance, and the endocrine system. Insulin resistance causes disorders such as obesity, dyslipidemia, hypertension, and metabolic syndrome in women with PCOS (2, 3). These side effects cause hyperandrogenemia, insulin resistance, inflammation, non-alcoholic liver damage, and damage to the cardiovascular system. It also induces metabolic changes in adipokines and inflammation triggered by adipose tissue (4, 5). Because of these effects, important tissues such as the ovary and liver cause disorders by releasing cytokines and reactive oxygen radicals (6, 7). Although there are many studies on PCOS, its pathophysiology remains unclear. Considering its pathophysiology, it may be associated with an increase in ROS and a corresponding decrease in anti-oxidant capacity. It also triggers inflammation due to increased ROS because of increased androgen and insulin resistance with PCOS (8, 9). In recent studies, various treatment methods have been tried to reduce the side effects of PCOS. These treatments include anti-oxidants, natural ingredients, flavonoids, and anti-inflammatory drugs (10-14). Although many treatments have been used to reduce the effect of PCOS, they were not sufficient to reduce the side effects and solve the mechanism of action. In particular, treatments to reduce the mechanism of action and side effects on the liver have been limited.

Astaxanthin (ASX), known as xanthine carotenoid, is a carotenoid found in *Haematococcus microalgae* (15). Studies have shown that ASX is a powerful anti-oxidant, especially by reducing lipid peroxidation and increasing SOD (16). Astaxanthin is a pigment with anti-oxidative, anti-inflammatory, antiapoptotic, anticancer, and immunomodulatory properties (17, 18). Current evidence indicates that AST has significant potential to inhibit the activation of several kinases, such as IKK kinase, during NF-kB activation, resulting in the blocking of its translocation to the nucleus via the dissociation of IKβ. In this way, several inflammatory-related genes interrupt their NF-kB-mediated expression. Blocking of NF-kB activation by ASX was the main reason explaining its anti-inflammatory effect (19). Significantly, some studies have shown that metformin, which is used for treating type 2 diabetes mellitus (DM), reduces oxidative stress. This drug increases anti-oxidant capacity by reducing ROS activation (20, 21). Co-administration of metformin and some natural anti-oxidants can prevent damage due to oxidative stress and insulin resistance (22). In this sense, it seems logical that ASX could increase the efficacy of metformin in reducing oxidative stress. In the studies conducted so far, the anti-oxidant effects of both drug combinations on the liver after PCOS have not been investigated. We conducted this study to determine the effects of astaxanthin, which is safe and cost-effective, and metformin, which is widely used in PCOS treatment, separately and in combination, on ROS production and cell death.

## Materiala and Methods

***Animals and experiment groups***

Fifty-four adult female Spraque-Dawley rats (weighing 200–250 g) obtained from Experimental Application and Research Center, Atatürk University, were used. The rats were fed a standard diet and kept in a physiological day-night rhythm. The study was approved by the Atatürk University Experimental Animal Research Ethics Committee (Approval number: no: 2021/2-35 ). Experimental rats were divided into 9 groups (n=6). The dose of administered letrozole, Met, and ASX was chosen according to previous studies (23-25).

The PCOS model and treatments applied are summarised in Table 1.

To confirm that PCOS was induced, vaginal smears were collected from the vagina of the rats with a plastic pipette filled with 10 µl of normal saline (NaCl 0.9%) at the same time each morning during the experiment and before the treatment. Estrus cycles were evaluated by examining exfoliated epithelial cells. After 21 days of letrozole gavage, it was observed that rats exhibited estrous cycle disorders and a prolonged diestrus phase (26, 27).

***Biochemical examination***

Liver tissue from the rats was stored at −80 °C and ground in liquid nitrogen using TissueLyser II (Qiagen, Hilden, Germany). Approximately 100 mg of powdered tissue was homogenized with 1 ml PBS. Samples were centrifuged for 15 min at 12000 rpm and 4 °C to assay superoxide dismutase (SOD) activity. SOD activity was measured using the method reported by Sun *et al.* (28). Homogenates were centrifuged at 4000 rpm for 15 min at 4 °C for malondialdehyde (MDA) activity analysis. Malondialdehyde (MDA) is a product of tissue lipid oxidation; It was measured using the method reported by Ohkawa *et al*. (29). All data were presented as mean±standard deviation results per mg protein.

***Immunohistochemical examination***

5 µm thick sections taken from paraffin blocks were stained using NF-kB (SC-8414; Santa Cruz, USA) and caspase 3 (PA5-77887; Thermo Scientific, USA) primary antibodies. Staining was performed using Ventana Benchmark Ultrasystem (Ventana Medical Systems, AZ, USA). The stained sections were examined under a light microscope (DP2-SAL firmware program; Olympus Inc., Tokyo, Japan) and photographed. Immunpositivity was evaluated for each section.

***Histological examination***

Liver tissues obtained from rats were fixed in 10% formaldehyde for 24 hr. They was then washed overnight in tap water to perform the routine histological follow-up procedure. Afterward, the blocks were prepared by passing through graded alcohol series, after the dehydration process, clearing with xylol, and infiltration with paraffin. Sections of 5 µm thickness were removed from the blocks by a microton and stained with Hematoxylin-Eosin (H&E) (30). Stained liver tissues were photographed and evaluated using the DP2-SAL firmware program and a light microscope (Olympus Inc., Tokyo, Japan). 

***Statistical analysis***

SPSS, version 20.0 (IBM Corp., Armonk, NY, USA) was used for statistical analysis. Additionally, results were presented as mean±standard deviation (S.D.) Statistical analysis of MDA, SOD, NF-kB, and caspase 3 was performed using one-way ANOVA and Duncan’s multiple comparisons test. A level with a *P*-value of <0.05 was considered significant.

## Results

***Biochemical results***

**Figure 1:**
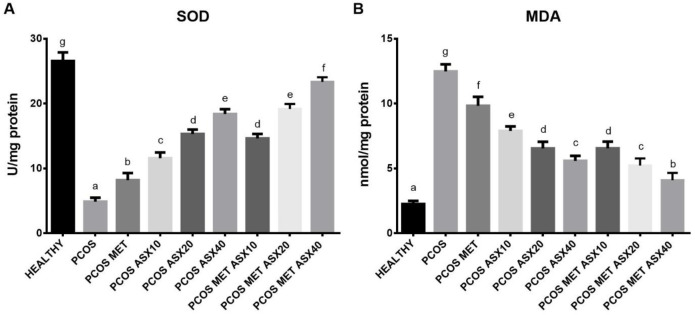
Effect of astaxanthin and metformin on malondialdehyde (MDA) and superoxide dismutase (SOD) levels in liver tissue. Groups: Healthy (Control), polycystic ovary syndrome (PCOS), PCOS+metformin (PCOS+Met), PCOS+ Astaxanthin 10 (PCOS+ASX10), PCOS+ Astaxanthin 20 (PCOS+ ASX20), PCOS+ Astaxanthin 40 (PCOS+ASX40), PCOS+ Metformin+ Astaxanthin 10 (PCOS+Met+ASX10), PCOS+ Metformin+ Astaxanthin 20 (PCOS+Met+ASX20), and PCOS+ Metformin+ Astaxanthin 40 (PCOS+Met+ASX40). All results are expressed as mean ± SD for each group. Comparisons were made between groups using One-way ANOVA and Duncan's test. Different letters (a,b,c..) show the statistical differences between the groups (*P*<0.05)

It was observed that tissue SOD levels of the PCOS group rats were significantly lower than those of the control group. PCOS+Met group SOD levels increased significantly compared with the PCOS group. However, PCOS+ASX groups had higher SOD levels than PCOS groups, depending on the dose. PCOS+Met group SOD levels were higher than the PCOS group, and PCOS+Met+ASX40 group SOD levels were significantly higher than those of other treatment groups (Figure 1A). However, the PCOS group exhibited a significantly higher MDA level than the control group. When the treatment groups were examined, it was observed that their MDA was significantly lower than that of the PCOS group. In particular, the PCOS+Met+ASX40 group MDA level, which is one of the combined treatment groups, was found to be closer to the control group, while it was significantly lower than the others (Figure 1B).

***Immunohistochemical results***

**Figure 2:**
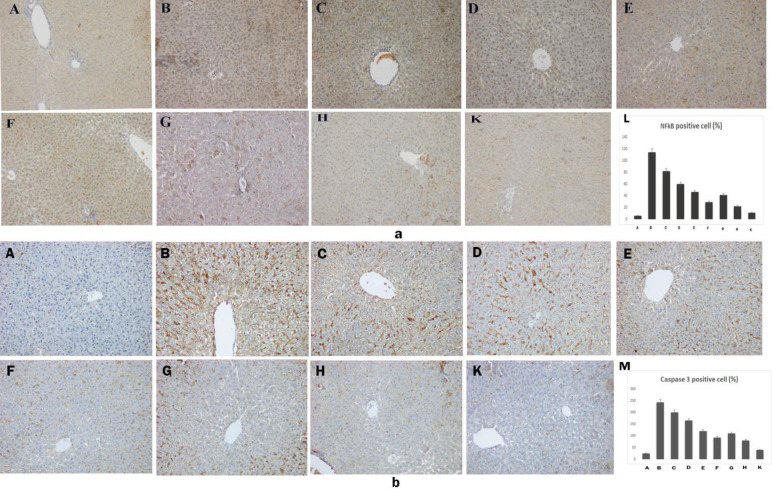
Immunohistochemistry of rat liver from experimental groups. (×20 magnifications). a: Nfkb staining. (A) Healthy, (B) PCOS, (C) PCOS+Met, (D) PCOS+ASX10, (E) PCOS+ASX20, (F) PCOS+ASX40, (G) PCOS+Met+ASX10, (H) PCOS+Met+ASX20, (K) PCOS+Met+ASX40, (L) NF-kB positive cell count

NF-kB immunohistochemical staining results are shown in Figure 2a. Compared with the control group, liver tissue NF-Kb immune positivity was significantly increased in the PCOS group (*P*<0.05). The PCOS+Met group had milder positive NF-kB expression than the PCOS model. A dose-dependent decrease in NF-Kb staining intensity was observed in the PCOS + ASX groups (*P*<0.05). In PCOS+Met+ASX groups, on the other hand, there was a decrease in NF-Kb staining intensity depending on the dose increase. In particular, NF-Kb immune positivity was significantly reduced in the PCOS+Met+ASX40 group.

When the image in Figure 2b was examined, it was observed that the caspase 3 immunohistochemical stained sections of the PCOS group were significantly higher than those of the control groups (*P*<0.05). When the image and score results of only PCOS+Met and only PCOS+ASX groups were examined, it was observed that caspase 3 immunopositivity was lower than that of PCOS. We observed that the liver tissue caspase 3 positive reaction in the PCOS+Met+ASX40 group was significantly lower than that in the other treatment groups. 

***Histological results***

**Figure 3:**
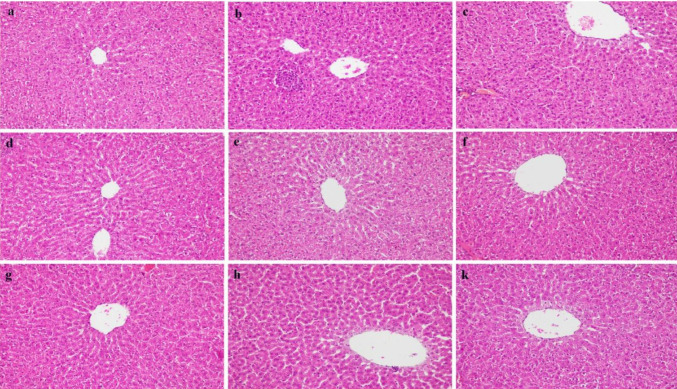
Photomicrographs of liver sections stained with Hematoxylin-Eosin. (×20 magnifications). (a) Healthy, (b) PCOS, white arrow: Infiltration of lymphocytes, white arrowhead: increased fat cells in liver tissue, curved arrow: necrosis, (c) PCOS+Met, white arrowhead: fat cells, curved arrow: necrosis, black arrow: congestion, black arrowhead: sinusoidal dilatation, (d) PCOS+ASX10, curved arrow: necrosis, (e) PCOS+ASX20, (f) PCOS+ASX40, (g) PCOS+Met+ASX10, (h) PCOS+Met+ASX20, (k) PCOS+Met+ASX40

Looking at the images in Figure 3, liver sections of the control group were observed in normal morphology (Figure 3a). When the rat livers of the PCOS group were examined, an increased inflammatory reaction and vacuole degeneration were observed in hepatocytes. Compared with the control, localized fat cell accumulations were observed. Necrotic areas were observed in some parts of the liver tissue (Figure 3b).  Sections in the PCOS+Met group showed necrotic hepatocytes in some places and mild vacuolar degeneration. Sinusoidal dilatations and congestion were observed in some regions of this group (Figure 3c). When the PCOS+ASX 10 group was examined, minimal improvements were observed in degenerative changes. Necrosis was observed in some hepatocyte cells in this group (Figure 3d). Central veins showed normal morphology in the PCOS+ASX 20 and PCOS+ASX 40 groups. Additionally, necrotic areas and dilatations decreased (Figure 3e, 3f). There was a significant decrease in inflammation and improvement in hepatic lesions, depending on the dose increase in the groups given metformin and astaxanthin-combined therapy. Hepatocyte cell borders were close to normal in the combined groups (Figure 3g, 3h,3k).

**Table 1:** Establishment of the polycystic ovary syndrome (PCOS) rat model and treatments

**Groups (** ***n*** ** = 6)**	**Considerations**
Group 1 (Control):	No treatment was applied to the animals in this group
Group 2 (PCOS)	1 mg/kg of letrozole was dissolved in 2 ml of carboxymethyl cellulose (CMC) and administered orally for 21 days
Group 3 (PCOS+Met)	20 mg/kg metformin was dissolved in 1 ml CMC and administered orally for 7 days to experimental animals with PCOS
Group 4 (PCOS+ASX10)	10 mg/kg ASX dissolved in 1 ml sesame oil was administered orally to rats with PCOS for 7 days
Group 5 ( (PCOS+ASX20)	20 mg/kg ASX dissolved in 1 ml sesame oil was administered orally to the experimental animals with PCOS for 7 days
Group 6 (PCOS+ASX40)	40 mg/kg ASX dissolved in 1 ml sesame oil was administered orally to the experimental animals with PCOS for 7 days
Group 7 (PCOS+Met+ASXl0)	Experimental animals with PCOS were given 20 mg/kg metformin in 1 ml CMC for 7 days; 10 mg/kg ASX was dissolved in 1 ml of sesame oil and administered orally
Group 8(PCOS+Met+ASX20)	Experimental animals with PCOS in the group were given 20 mg/kg metformin in 1 ml CMC for 7 days; 20 mg/kg ASX dissolved in 1 ml sesame oil and administered orally
Group 9 (PCOS+Met+ASX40):	Experimental animals with PCOS in the group were given 20 mg/kg metformin in 1 ml CMC for 7 days; 40 mg/kg ASX was dissolved in 1 ml of sesame oil and administered orally

b: caspase 3 staining. (A) Healthy, (B) PCOS, (C) PCOS+Met, (D) PCOS+ASX10, (E) PCOS+ASX20, (F) PCOS+ ASX40, (G) PCOS+Met+ASX10, (H) PCOS+Met+ASX20, (K) PCOS+Met+ASX40, (M) Caspase 3 -positive cell count. +++: strong positive staining, ++: mild positive staining, +: weak positive staining. All results are expressed as mean±SD for each group. Comparisons were made between groups using One-way ANOVA and Duncan's test. Different letters (a,b,c..) show the statistical differences between the groups (*P*<0.05)

## Discussion

PCOS is a common endocrine and metabolic disorder in fertile women. This syndrome is one of the main causes of infertility, affecting 6%–20% of women of reproductive age (31). In addition to infertility, PCOS increases the risk of obesity, type 2 diabetes, cancer, and cardiovascular disease (32). Although the etiology of PCOS is not well known, it is associated with insulin resistance, oxidative stress, and inflammation (13, 33).

Studies have shown that a significant proportion of patients with PCOS have elevated liver enzymes and increased non-alcoholic fatty liver disease (34). In previous PCOS-related liver studies, insulin resistance and obesity have been further evaluated (35). However, no clear mechanism could be reported for this condition. We used letrozole to create the PCOS model based on research and to mimic estrogen metabolism (36). Our histopathological results showed that we applied the PCOS model correctly. Additionally, we confirmed that cystic follicles were reduced by metformin, one of the PCOS treatment methods that have many examples in the literature. However, we have seen that application of this treatment alone is insufficient to reduce oxidative stress, inflammation, and apoptosis processes (37).

In the context of all this information, we investigated the effects of astaxanthin, an effective anti-oxidant, on the liver tissues of PCOS model-induced rats in the current study. In our study, we evaluated the effect of PCOS in terms of many parameters. Oxidative stress is known to be an important factor in the pathophysiology of PCOS. Studies have shown that increased oxidative stress is associated with high androgen production and polycystic ovary syndrome (38-40).

Additionally, studies have revealed that oxidative stress may play an important role in different types of damage in patients with PCOS (41-43). Researchers emphasized that ROS, a natural byproduct of normal oxygen metabolism, plays an important role in cell signaling mechanisms and homeostasis in the body, but that its production at abnormally high levels in patients with PCOS is due to the imbalance between oxidation and anti-oxidation (44). MDA, which is one of the important markers of oxidative stress and shows lipid peroxidation, is critical to toxicity and cell death (45). In this study, we found that there was a significant increase in MDA level in the liver tissue of rats treated with the PCOS model compared with the control group. Our findings were in parallel with the findings in the PCOS study of Ghowski *et al*. (7). Studies have reported that this significant increase in MDA level may be associated with both an increase in androgen production and insulin resistance (46, 47).

 There was a decrease in MDA level compared with PCOS only in the group that received metformin after PCOS. In this study, we found that the MDA level decreased in the groups given only astaxanthin compared with the PCOS group, depending on the dose. Administration of metformin and increasing doses of astaxanthin after PCOS further decreased MDA levels. With this result, we thought that astaxanthin increased the anti-oxidant level and thus decreased the increased ROS. In different toxicity models made before, it has been shown that astaxanthin reduces oxidative stress (16, 48). These findings are consistent with our results. Our results show that an excessive increase in PCOS-induced ROS level causes liver damage. Oxidative stress, apoptosis, inflammation, DNA breaks, and lipids play a major role in the initiation of this damage. One of the important mechanisms caused by oxidative stress is the depletion of anti-oxidants. In previous studies, a decrease in anti-oxidant levels was observed in patients with PCOS (40, 42, 46). Our findings are consistent with these results. In this study, SOD levels decreased in the PCOS group compared with the control group.

SOD catalyzes the dismutation of superoxide and the detoxification of H_2_O_2_. SOD levels were increased in the treatment group given together with metformin compared with the letrozole group in the current study. These results were in agreement with previous studies. (37, 40, 43) We concluded that astaxanthin given for treatment may be beneficial against decreased anti-oxidant capacity and harmful effects of increased oxidative stress in patients with PCOS. Another cause of oxidative stress is inflammation. NF-kB, which plays an important role in inflammation, is phosphorylated and deactivated by IkBa and IkB kinase-β (IKKβ) at any time of damage. In this way, it allows the release of proinflammatory cytokines (49, 50). In this study, we found that metformin and astaxanthin together reduced NFκB expression. This result revealed that astaxanthin given for treatment is anti-inflammatory. As a result of pathological changes in patients with PCOS, the apoptosis process begins. Mitochondrial changes begin in hepatocyte cells due to stress, and as a result, apoptotic pathways are activated. Among these pathways, the level of caspase 3 may increase because of oxidative stress (30, 50). In this study, when the results of the immunohistochemical staining and score table were examined, it was seen that the caspase 3 level was higher in the PCOS group than in the other groups. These results were found to be in agreement with the studies in the literature** (**50, 51). There was a decrease in caspase 3 levels in the metformin-administered groups. However, it was observed that the combined administration of two treatment drugs was more effective depending on the dose increase. We observed that the Caspase 3 level decreased more in the combined groups. When we looked histologically, we saw that the liver tissue of the control group was in normal morphology. However, when the PCOS group was examined, the increase in inflammatory cells was remarkable. We also observed local fat accumulation and necrotic areas in rats. Histological differences were observed in accordance with the studies in the literature (52, 53). When we looked at the metformin group, we observed locally necrotic hepatocytes and mild vacuolar degeneration. However, a decrease in inflammation was observed in the group given metformin and astaxanthin together. Additionally, there was a decrease in hepatic lesions.

## Conclusion

According to our results, we found that PCOS disease increases lipid peroxidation in the liver, resulting in oxidative stress. Likewise, we saw that it increased the levels of NF-kB and caspase 3. This situation can cause oxidative stress, apoptosis in liver diseases, and necrotic tissue damage. Based on these results, we found that administration of astaxanthin improves oxidative stress, inflammation, apoptosis, and tissue pathological changes. However, we saw that the results of administration of metformin and astaxanthin were more positive. However, since our possibilities are limited, we think that it would be beneficial to study both the model and some damage pathways in detail.

## Authors’ Contributions

 TBT, NA, ÖD, and RAU designed and performed the research, analyzed data, and wrote and edited the article.

## Conflicts of Interest

The authors of this manuscript have no conflicts of interest to declare.
